# C1q/TNF-Related Protein5 (CTRP5) as a Biomarker to Predict Metabolic Syndrome and Each of Its Components

**DOI:** 10.1155/2018/7201473

**Published:** 2018-11-22

**Authors:** Feiyu Jiang, Min Yang, Xili Zhao, Rui Liu, Gangyi Yang, Dongfang Liu, Hua Liu, Hongting Zheng, Zhiming Zhu, Ling Li

**Affiliations:** ^1^Key Laboratory of Diagnostic Medicine (Ministry of Education) and Department of Clinical Biochemistry, College of Laboratory Medicine, Chongqing Medical University, 400016, China; ^2^Department of Endocrinology, The Second Affiliated Hospital, Chongqing Medical University, Chongqing, China; ^3^Department of Pediatrics, University of Mississippi Medical Center, 2500 North State Street, Jackson, Mississippi, USA; ^4^Department of Endocrinology, Xinqiao Hospital, Third Military Medical University, Chongqing, China; ^5^Department of Hypertension and Endocrinology, Daping Hospital, Third Military Medical University, Chongqing Institute of Hypertension, Chongqing, China

## Abstract

**Objective:**

C1q/TNF-related protein5 (CTRP5) is a member of the C1q/tumor necrosis factor *α*- (TNF-*α*-) related protein family and has been reported to be associated with the regulation of glucose and lipid metabolism. However, the clinical association between CTRP5 and metabolic syndrome (MetS) has not been reported. The aim of the current study is to investigate the association between CTRP5 and MetS by a cross-sectional study.

**Methods:**

We performed a cross-sectional study in a Chinese population including 89 controls and 88 MetS individuals. Serum CTRP5 concentrations were determined by ELISA. The relationship between circulating CTRP5 and MetS and insulin resistance (IR) was assessed by Spearman's correlation and multiple stepwise regression analysis.

**Results:**

Circulating CTRP5 concentrations were markedly decreased in MetS individuals relative to normal adults. Overweight/obese individuals (BMI ≥ 25 kg/m^2^) showed a lower serum CTRP5 level than lean subjects (BMI < 25 kg/m^2^) in the study population (124.1 (99.12–147.37) vs. 103.9 (79.15–124.25) *μ*g/L; *P* < 0.01). Circulating CTRP5 was found to be correlated negatively with BMI, FAT%, FBG, WHR, SBP, HbA1c, TG, 2-hour blood glucose after glucose overload (2-hOGTT), FIns, and HOMA-IR and positively with HDL-C (*P* < 0.05 or *P* < 0.01). Binary logistic regression revealed that serum CTRP5 levels were associated with MetS. In addition, serum CTRP5 levels gradually decreased with the increase in MetS components.

**Conclusions:**

Circulating CTRP5 is relative to the elevated risk of MetS in humans and may be in part through the effect of insulin resistance. This trial is registered with ChiCTR-OCS-13003185.

## 1. Introduction

Metabolic syndrome (MetS) consists of a group of metabolic risk factors, including hypertension, central obesity, insulin resistance (IR), and dyslipidemia [1–4]. Numerous studies have shown that MetS has become a worldwide public health problem, which directly increases the prevalence of cardiovascular disease (CVD) [5], cancer [6, 7], type 2 diabetes mellitus (T2DM), and all-cause mortality [8]. IR is known to be the main feature of MetS [9], although its exact mechanism has not been yet clear.

Adiponectin, which is mainly derived from adipose tissue, is an insulin-sensitizing hormone [10]. Some studies have shown that MetS subjects have lower serum adiponectin (Adipq) levels. In addition, circulating Adipq levels are inversely associated with IR [11, 12]. C1q/TNF-*α*-related protein isoform 5 (CTRP5) has been identified as an adiponectin paralogue and is mainly expressed in adipose tissue, which shares functional and structural similarities to adiponectin [13–19]. A polymorphism in the CTRP5 gene has been found to be related to the development of MetS in a Japanese population by a genome-wide association study (GWAS) [20]. Several studies have shown that CTRP5 is related to the regulation of lipid and glucose metabolism [13, 21]. Park et al. revealed that serum CTRP5 is elevated in animals with insulin resistance (IR) [13] and CTRP5 promoted the phosphorylation of AMP-activated protein kinase (AMPK), increased glucose uptake by promoting the translocation of glucose transporter-4 (GLUT4) in the plasma membrane, and elevated the phosphorylation of acetyl-CoA carboxylase to stimulate fatty acid oxidation and insulin sensitivity [13]. Animal studies have demonstrated that circulating CTRP5 levels are higher in obese and IR mice [13]. However, in human studies, the results have been inconsistent. It has been found that in nonalcoholic fatty liver disease (NAFLD) and T2DM patients, serum CTRP5 levels were lower compared with those in healthy controls [22], whereas Park et al. found that the expression and secretion of the CTRP5 gene were increased in myocytes of IR individuals due to mitochondrial dysfunction [13]. Recently, it has been reported that insulin-stimulated phosphorylation of Akt, an important component of insulin signaling pathways, in adipocytes and myotubes was attenuated by CTRP5 protein treatment [23]. However, the clinical association between circulating CTRP5 and MetS has not been reported. In our cross-sectional study, serum levels of CTRP5 in obese and MetS individuals were investigated and the relationship between CTRP5 and the pivotal components of MetS in a Chinese population was analyzed.

## 2. Materials and Methods

### 2.1. Study Subjects

A total of 177 individuals aged 41–70 yr (89 healthy controls and 88 MetS subjects) were recruited for the current study from the community through advertisement or routine medical check-up in the Department of Endocrinology at the Second Affiliated Hospital of Chongqing Medical University between 2016 and 2017. The diagnosis of MetS was based on the United States National Cholesterol Education Program (NCEP) Expert Panel Adult Treatment Panel III (ATP III) [4] and modified as recommended in the latest American Heart Association/National Heart, Lung and Blood Institute Scientific Statement by adopting the Asian criteria for waist circumference (WC) and a lower cutoff for fasting blood glucose (FBG) [2]. MetS was defined as having three or more of the following metabolic risk factors: (1) central obesity (WC ≥ 80 cm in females and ≥90 cm in males), (2) hypertriglyceridemia (triglyceride (TG) ≥ 1.69 mmol/L)), (3) high-density lipoprotein cholesterol (HDL < 1.29 mmol/L in females and <1.04 mmol/L in males), (4) hyperglycemia (FBG ≥ 5.6 mmol/L or T2DM), and (5) hypertension (sitting blood pressure (BP) ≥ 130/85 mmHg, taken as a mean of two readings obtained after resting for at least 10 min or receiving antihypertensive medication). Exclusion criteria include individuals with cancer, liver cirrhosis, active infection, heart failure, long-term treatment of steroid, or other medical problems. All MetS patients had not been treated with any agents and were newly diagnosed. All control subjects had no clinical evidence of major diseases. We obtained written voluntary informed consent from all subjects before they participated in the study. The present study was conducted in accordance with the Declaration of Helsinki and approved by the human research ethics committee of Chongqing Medical University and was registered at ChiCTR-OCS-13003185.

### 2.2. Anthropometric and Biochemical Measurement

Anthropometric measurements were performed in all study participants before breakfast. Weight and height were obtained by trained physicians, with participants wearing light indoor clothing and barefooted, using calibrated portable electronic weighing scales. Body mass index (BMI) was calculated as weight (kilograms) divided by squared height (meters). The same observer used an inelastic measuring tape on the bare skin to measure WC and hip circumference (HC) which was recorded to the nearest 0.1 cm. Waist-to-hip ratio (WHR) was calculated as WC/HC. BP was measured on the nondominant arm using a mercury sphygmomanometer after at least 10 min rest. The average of two measurements taken with a 5–10-minute interval was used for analysis. We used bioelectrical impedance (BIA-101; RJL Systems, Shenzhen, China) to examine the percentage of the body fat (FAT%). After 10–12 hr fast, venous blood was collected and centrifuged to separate the serum. Samples were stored at −80°C for subsequent analysis. Blood glucose and HbA1c were immediately measured by the glucose-oxidase method and anion-exchange HPLC, respectively.

Low-density lipoprotein cholesterol (LDL-C), high-density lipoprotein cholesterol (HDL-C), triglyceride (TG), and total cholesterol (TC) were determined enzymatically (Randox Laboratories Ltd., Antrim, UK, CH8311-12, TR9780, CH8019). Fasting insulin (FIns) levels were measured by an electrochemiluminescence method (Roche Diagnostics GmbH, Basel, Switzerland, 29701202). Free fatty acids (FFAs) were determined by using a commercial kit (Randox Laboratories Ltd., Antrim, UK, FA115). The homeostasis model assessment of IR (HOMA-IR) was calculated by the following equation: HOMA − IR = FIns (mU/L) × fasting blood glucose (FBG, mmol/L)/22.5 [24].

### 2.3. Measurement of Circulating CTRP5 Concentration

Serum CTRP5 concentrations were measured using ELISA kits obtained from Aviscera Bioscience Inc. (CA, USA, SK00594-09, 20112888). The intra- and interassay coefficients of variance (CV) were 6–8% and 8–12%, respectively. Linearity was in the range of 1.56–200 *μ*g/L. The limit of detection for this assay was 0.2 *μ*g/L. In addition, a spike recovery test was performed to confirm the efficacy of the ELISA kit (data not shown).

### 2.4. Statistical Analysis

All analyses were performed with Statistical Package for the Social Sciences version 19.0 (SPSS Inc., Chicago, IL). Normally distributed data were expressed as mean ± SD. Data of nonnormal distribution were skewed and logarithmically transformed to obtain a normal distribution, which were expressed as median with interquartile range (IQR). The unpaired *t*-test or one-way ANOVA was performed to analyze the differences of two or more groups. Spearman's correlation analysis was used to examine the association of circulating CTRP5 with other parameters. Relationships between the CTRP5 and the other variables were investigated by using multiple stepwise regression analysis with CTRP5 as a dependent variable. Multivariate logistic regression analysis was used to investigate the association of CTRP5 with MetS. Receiver operating characteristic (ROC) curves were used to analyze the predicting values of circulating CTRP5 for MetS and IR. All data were based on two-sided tests. *P* < 0.05 was considered statistically significant.

## 3. Results

### 3.1. Characteristics of the Study Population

The anthropometric and biochemical parameters of the studied population are shown in Table 1. There was no significant difference in the sex ratio between healthy controls and MetS subjects (male/female: 37/52 for controls and 35/53 for MetS subjects). However, MetS individuals had higher BMI, FAT%, FBG, WHR, BP, HbA1c, TG, 2-hour blood glucose after glucose overload (2-hOGTT), FIns, and HOMA-IR (*P* < 0.05 or *P* < 0.01) and lower HDL-C levels (*P* < 0.01). After adjustment for age and sex, these differences still existed except for diastolic blood pressure (DBP).

### 3.2. Circulating CTRP5 Levels and Its Association with Other Parameters

**Figure 1:**
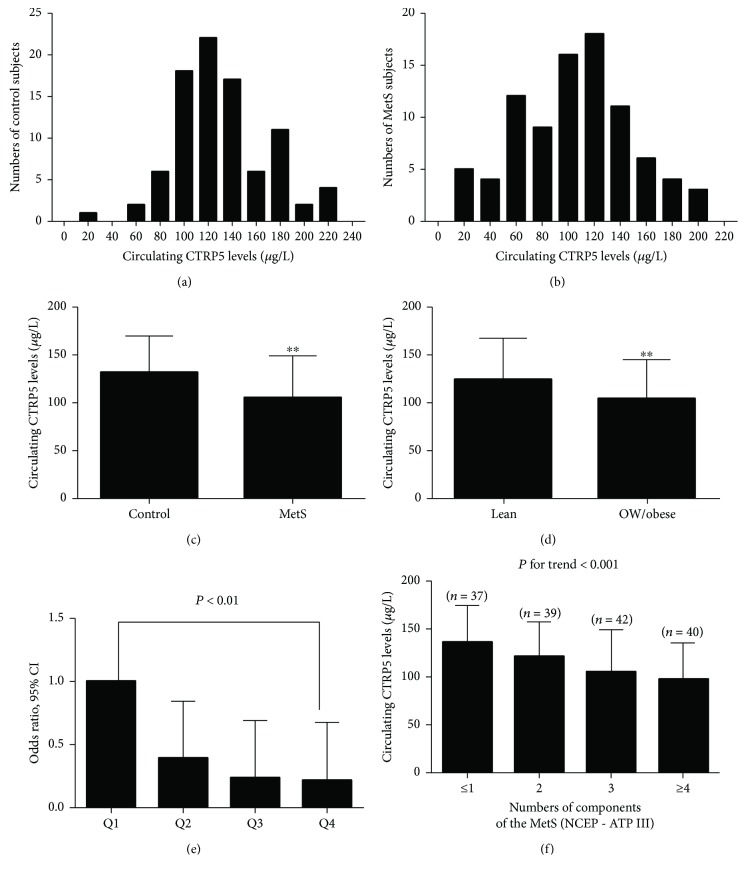
Circulating CTRP5 levels in the study population: (a) distribution of circulating CTRP5 levels in 89 control subjects; (b) distribution of circulating CTRP5 levels in 88 MetS subjects; (c) circulating CTRP5 levels in control and MetS subjects (^∗∗^*P* < 0.01 compared with controls); (d) circulating CTRP5 levels in all studied population according to BMI (lean: BMI < 25 kg/m^2^ and OW/obese: BMI ≥ 25 kg/m^2^; ^∗∗^*P* < 0.01 compared with lean subjects); (e) odds ratio (OR) for having MetS according to the quartiles of circulating CTRP5 levels (reference, the lowest quartile) (OR: odd ratio; Q: quartile); (f) circulating CTRP5 levels decreased progressively with increasing numbers of components of MetS. *P* < 0.05 was considered significant.

Fasting CTRP5 was examined in 177 adults aged 41–70 yr in this study. The distributions of CTRP5 levels in these individuals are shown in Figure 1(a) for normal adults and Figure 1(b) for MetS subjects. The range of CTRP5 levels was 19.81–226.54 *μ*g/L for normal subjects and 11.00–203.37 *μ*g/L for MetS subjects. CTRP5 levels were between 56.25 and 206.33 *μ*g/L for 95% healthy subjects and 18.19 and 191.69 *μ*g/L for 95% MetS subjects. Importantly, serum CTRP5 concentrations were markedly decreased in MetS individuals relative to normal adults (Table 1 and Figure 1(c)). After adjustment for age, sex, and BMI, this difference remained significant. In addition, we stratified the recruited subjects into two groups according to their BMI levels; overweight/obese individuals (BMI ≥ 25 kg/m^2^) showed a lower serum CTRP5 level than lean subjects (BMI < 25 kg/m^2^) (124.1 (99.12–147.37) vs. 103.9 (79.15–124.25) *μ*g/L; Figure 1(d), *P* < 0.01). However, there was no gender difference in the circulation levels of CTRP5 in the control group and MetS group (data were not shown), indicating that CTRP5 has no distinct sexual dimorphism. Then, we performed partial correlation analysis to explore the relationship between serum CTRP5 concentrations and other parameters. Circulating CTRP5 correlated negatively with BMI, FAT%, FBG, WHR, SBP, HbA1c, TG, 2-hour blood glucose after glucose overload (2-hOGTT), FIns, and HOMA-IR in the whole population but positively with HDL-C (Table 2, *P* < 0.05 or *P* < 0.01). There was no relationship between circulating CTRP5 and TC and LDL-C. In multiple stepwise regression analysis, we found that the main determinants of circulating CTRP5 were HOMA-IR and WHR. The beta-coefficients for HOMA-IR and WHR were −0.50 and −0.16, respectively. The multiple regression equation is *Y*_CTRP5_ = 2.750 − 0.019 × log HOMA − IR − 0.570 × WHR (*P* < 0.01, *R*^2^ = 0.344).

### 3.3. The Association of CTRP5 with MetS Components and Its Predictive Value for MetS and IR

**Figure 2:**
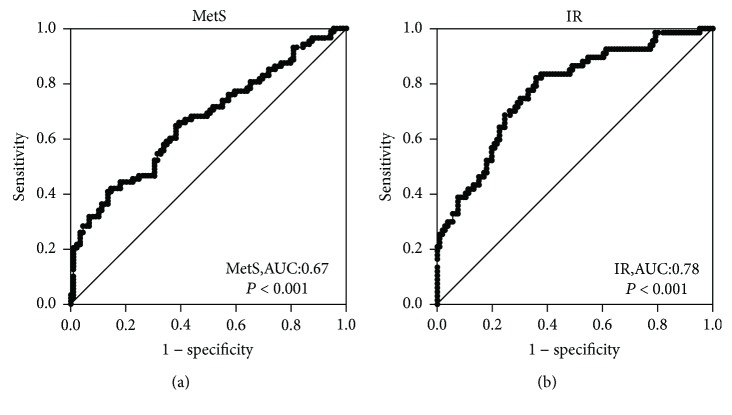
ROC curve analyses were performed for the prediction of MetS (a) and IR (b).

To further investigate the association between CTRP5 and MetS, we performed binary logistic regression. The result showed that the association between CTRP5 and MetS remained significant even after controlling for anthropometric variables and lipid profile (Table 3). To further investigate the relationship between CTRP5 and MetS, CTRP5 levels were divided into four quartiles according to its concentration in the study (quartile 1, <92.94 *μ*g/L; quartile 2, 92.94–116.94 *μ*g/L; quartile 3, 116.94–145.14 *μ*g/L, and quartile 4, >145.14 *μ*g/L), and then logistic regression analysis was performed to calculate the odds of having MetS. When CTRP5 levels were in quartile 4, the odds ratios of having MetS were 0.21 (vs. quartile 1, *P* < 0.01; Figure 1(e)). Furthermore, we stratified the concentrations of serum CTRP5 according to the number of MetS components. We found that CTRP5 levels gradually decreased with the increase in the MetS components (Figure 1(f)). Finally, we performed ROC curve analysis of serum CTRP5 for predicting MetS and IR. The area under the ROC curves (AUC_ROC_) was 0.67 (*P* < 0.001) with a sensitivity of 61% and a specificity of 66% for detecting MetS (Figure 2(a)) and 0.78 (*P* < 0.001) with a sensitivity of 64% and a specificity of 82% for IR (Figure 2(b)). The best cutoff values for CTRP5 to predict MetS and IR were 118.67 and 119.99 *μ*g/L, respectively.

## 4. Discussion

Although there are reports in which circulating CTRP5 levels were shown to be low in NAFLD and T2DM patients [22], there have been no reports on the circulating levels of this cytokine in MetS subjects. In the current study, for the first time, we find that serum CTRP5 levels were reduced in MetS patients relative to normal adults. Our results were contrary to an animal study, which reported an increase in serum CTRP5 levels in obese or diabetic mice [13]. However, the results were similar to a human study, which reported a decrease in circulating CTRP5 concentration in NAFLD and diabetic patients relative to normal individuals [22]. The cause of the discrepancy between animals and humans is unknown. Notably, a discrepancy has also been found in other CTRP members such as CTRP3 and CTRP1 [14, 25, 26]. We speculate that the role of this cytokine is not exactly the same in human and mice, as resistin has different roles in human and mice [27]; (2) It is also likely that there are differences between animals and patients with T2DM or MetS. Animal models may only reflect certain aspects of these diseases. For instance, some animal models mainly expressed IR, while others mainly manifested *β*-cell failure [28]. To clarify this problem, further studies are needed.

CTRP5 has been shown to be an important secretion protein related to obesity [18]. Therefore, in the current study, we also analyze the relationship between CTRP5 and obesity as well as other anthropologic and metabolic parameters. Our data reveals that serum CTRP5 concentrations were significantly lower in overweight/obese individuals compared with lean subjects. There was a negative correlation between CTRP5 and obesity-related indicators, including FAT%, WHR, and TG. These results further indicate that CTRP5 might act as a serum biomarker for obesity and related metabolic diseases, such as T2DM and MetS. In fact, CTRP5 has been identified as a candidate gene of obesity in humans by a microarray gene profiling study [29]. However, in a study of a Korean population, Choi et al. reported that circulating CTRP5 concentrations were not significantly different in obese and lean subjects, and circulating CTRP5 values were not related to the markers of adiposity [30]. The cause of the discrepancy between the previous results and those of the current study is unclear. However, these discrepancies could be attributed to (1) the different clinical characteristics of the two study cohorts, including age, percentage of males and females, and difference in BMI; (2) differences of the criteria adopted for obesity and MetS; (3) the heterogeneity of the study population, such as the level of physical activity of the subjects and pharmacotherapy; and (4) the difference in measurement, which may provide different results for CTRP5 levels. Because the study in vitro has shown beneficial effects of CTRP5 on insulin sensitivity, such as inducing phosphorylation of AMP-activated protein kinase (AMPK), thereby stimulating glucose uptake and fatty acid oxidation [13], the decreased CTRP5 levels in obese or MetS subjects might be due to the consumption by the human body to counteract the metabolic stress or an inflammatory state characterized by dysregulated production of adipokines. However, the precise mechanisms that drive the relationship between CTRP5 and metabolic derangements are not clear. Further studies are needed.

In the study population, when serum CTRP5 was divided into different concentrations, the prevalence of MetS was increased progressively with reduced CTRP5 concentrations. In addition, our results also showed a relationship between circulating CTRP5 and the components of MetS. When circulating CTRP5 was in low concentrations, the number of MetS components, including WC, BP, blood glucose, and TG, increased. When circulating CTRP5 concentrations were higher than 140 *μ*g/L, individuals were more likely to demonstrate only one component of MetS, although it is not possible to say that these individuals were metabolically healthy.

In our multiple linear regression analysis, we found that HOMA-IR and WHR were independent contributors of circulating CTRP5. Furthermore, binary logistic regression analysis showed that CTRP5 concentrations were significantly associated with MetS. The evidence of this cytokine shows the importance of using it as a biomarker for MetS.

Finally, ROC curve analysis showed that the AUC values for MetS and IR indicate that circulating CTRP5 has moderate diagnostic capabilities in identifying subjects with MetS or IR. ROC curve analysis also showed a potential for determining cutoff points for circulating CTRP5 to detect MetS. In addition, correlation analysis showed that circulating CTRP5 was closely related to metabolic risk factors for MetS, including obesity, hypertension, hyperglycemia, and dyslipidemia. We therefore believe that the detection of circulating CTRP5 is of great significance for screening MetS. To determine the existence of IR, the euglycemic-hyperinsulinemic clamp (EHC) is considered a “gold standard” measure of insulin sensitivity [31]. However, it is not available in a clinical setting and is costly, time consuming, and invasive and requires trained staff. Therefore, finding a good predictor of IR is important. We thus propose whether CTRP5 can be used as a primary screening index for IR. However, further longitudinal studies should be carried out to better understand the role of CTRP5 in the pathogenesis of MetS and IR.

This study has some limitations due to its nature of cross-sectional design. First, it is impossible to establish the cause-effect association. Second, we were unable to determine which of the MetS components had a greater effect on circulating CTRP5 concentrations. Third, the predictive significance of CTRP5 as a biomarker in MetS subjects remains to be confirmed by prospective and clinical validation studies. Further in-depth study should be performed to identify the cause-effect association between CTRP5 and MetS. In addition, our samples are entirely from Chinese people. Therefore, these results should be carefully extrapolated to other ethnic groups. However, the strength of the current study is that it compares the predictive power of circulating CTRP5 while providing cutoff values for the prediction of MetS in a Chinese population and it utilizes the appropriate method (ELISA) for circulating serum CTRP5 measurement.

In conclusion, the current study shows a novel result in that the low levels of serum CTRP5 were associated with MetS and IR in adults. Circulating CTRP5 levels gradually decreased with the increase in the MetS components. Our results suggest that CTRP5 may be used as a serum biomarker for obesity-related diseases, such as MetS and T2DM as well as cardiovascular disease. However, further in-depth study is required to investigate whether CTRP5 could be a novel therapeutic target for MetS and IR.

## Figures and Tables

**Table 1:** Anthropometric and biochemical parameters in MetS and healthy subjects.

Variables	Control (*n* = 89)	MetS (*n* = 88)	*P*		
			Not adjusted	Adjusted for age and sex	Adjusted for age, sex, and BMI
Sex (male/female)	37/52	35/53	NS		
Age (year)	54 ± 12	56 ± 9	<0.05		
BMI (kg/m^2^)	22.54 ± 2.29	25.01 ± 2.68	<0.01	<0.01	
FAT%	28.1 (23.9–31.7)	31.2 (25.8–35.9)	<0.01	<0.01	NS
WHR	0.87 ± 0.06	0.92 ± 0.05	<0.01	<0.01	NS
SBP (mmHg)	117.6 ± 13.3	128.1 ± 11.5	<0.01	<0.01	<0.01
DBP (mmHg)	74.5 ± 9.5	77.7 ± 9.4	<0.05	NS	NS
TG (mmol/L)	1.10 (0.92–1.40)	1.50 (1.04–2.64)	<0.01	<0.01	<0.01
TC (mmol/L)	4.81 ± 0.83	4.98 ± 0.94	NS	NS	NS
HDL-C (mmol/L)	1.40 ± 0.25	1.22 ± 0.28	<0.01	<0.01	<0.01
LDL-C (mmol/L)	2.82 ± 0.77	2.76 ± 0.82	NS	NS	NS
FBG (mmol/L)	5.34 (4.99–6.21)	7.44 (5.90–9.53)	<0.01	<0.01	<0.01
2-hOGTT (mmol/L)	6.75 (5.64–11.05)	11.67 (7.56–14.99)	<0.01	<0.01	<0.05
FIns (mU/L)	8.24 (6.03–12.04)	12.13 (9.09–17.89)	<0.01	<0.01	<0.01
HbA1c (%)	5.55 (5.30–7.00)	7.45 (6.07–8.50)	<0.01	<0.01	<0.01
HOMA-IR	2.05 (1.44–3.10)	4.39 (3.05–6.84)	<0.01	<0.01	<0.01
CTRP5 (*μ*g/L)	125.3 (105.3–154.8)	109.1 (71.1–133.2)	<0.01	<0.01	<0.05

Data are shown as means ± SD or median (interquartile range). MetS: metabolic syndrome; NS: not significant; BMI: body mass index; FAT%: the percentage of body fat; WHR: waist-to-hip ratio; SBP: systolic blood pressure; DBP: diastolic blood pressure; TG: triglyceride; TC: total cholesterol; HDL-C: high-density lipoprotein cholesterol; LDL-C: low-density lipoprotein cholesterol; FBG: fasting blood glucose; 2-hOGTT: 2-hour blood glucose after glucose overload; FIns: fasting insulin; HbA1c: glycated hemoglobin A1c; HOMA-IR: the homeostasis model assessment of insulin resistance.

**Table 2:** Partial correlations of circulating CTRP5 levels with anthropometric and biochemical parameters.

Variables	CTRP5^∗^		CTRP5^∗^ (age-adjusted)		CTRP5^∗^ (age- and BMI-adjusted)	
	*r*	*P*	*r*	*P*	*r*	*P*
BMI (kg/m^2^)	−0.297	<0.01	−0.163	NS		
FAT%	−0.167	<0.05	−0.144	NS	0.106	NS
WHR	−0.266	<0.01	−0.217	<0.05	−0.176	<0.05
SBP (mmHg)	−0.196	<0.05	−0.108	NS	−0.024	NS
TG^∗^ (mmol/L)	−0.184	<0.05	−0.184	<0.05	−0.106	NS
TC (mmol/L)	−0.144	NS	−0.068	NS	−0.076	NS
HDL-C (mmol/L)	0.114	<0.05	0.102	<0.05	0.072	NS
LDL-C (mmol/L)	−0.075	NS	−0.053	NS	−0.059	NS
FBG (mmol/L)	−0.412	<0.01	−0.314	<0.01	−0.280	<0.01
2-hOGTT	−0.377	<0.01	−0.315	<0.01	−0.288	<0.01
FIns^∗^ (mU/L)	−0.418	<0.01	−0.425	<0.01	−0.382	<0.01
HbA1c (%)	−0.396	<0.01	−0.338	<0.01	−0.306	<0.01
HOMA-IR^∗^	−0.484	<0.01	−0.450	<0.01	−0.427	<0.01

^∗^Log-transformation; NS: not significant; BMI: body mass index; FAT%: the percentage of body fat; WHR: waist-to-hip ratio; SBP: systolic blood pressure; TG: triglyceride; TC: total cholesterol; HDL-C: high-density lipoprotein cholesterol; LDL-C: low-density lipoprotein cholesterol; FBG: fasting blood glucose; 2-hOGTT: 2-hour blood glucose after glucose overload; FIns: fasting insulin; HbA1c: glycated hemoglobin A1c; HOMA-IR: the homeostasis model assessment of insulin resistance.

**Table 3:** Association of circulating CTRP5 with MetS in fully adjusted models.

	MetS		
	OR	95% CI	*P*
Age, SBP, DBP	0.985	0.976–0.994	<0.01
Age, SBP, DBP, BMI, WHR	0.990	0.980–1.000	<0.05
Age, SBP, DBP, BMI, WHR, lipid profile	0.987	0.975–1.000	<0.05

Results of multivariate binary logistic regression analysis are presented as the odds ratio (OR) for having a decreased MetS status in circulating CTRP5.

## Data Availability

The datasets generated and/or analyzed during the current study are available from the corresponding author on reasonable request.
